# The Role of Machine Learning Models in Predicting Cirrhosis Mortality: A Systematic Review

**DOI:** 10.7759/cureus.78155

**Published:** 2025-01-28

**Authors:** Khadija Abdikadir Mohamud, Suha Abdelhai Elzubair Eltahir, Hind AbdAlla Ahmed Alhardalo, Hadel Bakhet Albashir, Nora Qassem Alsyed Ali Mohamed Zain, Mohamed Elsayed Abdelrahman Ibrahim, Enas Nusreldeen Ahmed Fadlallah

**Affiliations:** 1 Faculty of Medicine, International University of Africa, Khartoum, SDN; 2 Department of Internal Medicine, Dr. Sulaiman Al Habib Medical Group Hospital, Buraydah, SAU; 3 Department of General Medicine, Abu Dhabi Health Services Company (SEHA) - Salma Rehabilitation Hospital, Abu Dhabi, ARE; 4 Department of General Practice, Abu Dhabi Health Services Company (SEHA) - Salma Rehabilitation Hospital, Abu Dhabi, ARE; 5 Department of Internal Medicine, Najran Armed Forces Hospital, Najran, SAU; 6 Department of Chemical Pathology, Aberdeen Royal Infirmary Foresterhill Health Campus, Aberdeen, GBR

**Keywords:** artificial intelligence, end-stage liver disease, liver cirrhosis, machine learning, predictive model

## Abstract

Liver cirrhosis affects millions of individuals worldwide and is one of the primary causes of mortality. Early mortality prediction for cirrhosis patients may increase the possibility for medical professionals to treat the illness successfully. This study assesses the ability of machine learning (ML) models to predict cirrhosis mortality. We followed Preferred Reporting Items for Systematic Reviews and Meta-Analyses (PRISMA) guidelines to search for relevant literature across four different databases. We found 379 studies of which 10 were eligible for inclusion in the current study. We analyzed 10 retrospective studies that showed that ML models outperformed conventional scores in predicting the death rate from end-stage liver disease (ESLD). Interestingly, models that used more parameters, such as patient demographics and extensive laboratory testing, exhibited higher prediction accuracy. With an area under the receiver operating characteristic (AUROC) ranging from 0.71 to 0.96, ML models showed consistently significant gains over traditional prognostic ratings. This review emphasizes how ML models might improve ESLD patient death prediction. Because machine learning models are more accurate than conventional approaches, it is important to incorporate data-driven informatics technologies into clinical settings. Additional validation and openness are required to guarantee model dependability and interpretability before ML may be used in clinical practice. The goal of future research should be to create reliable, interpretable models that may be used successfully in a variety of clinical contexts, enhancing ESLD patient treatment and results.

## Introduction and background

Liver cirrhosis is a major cause of death and disease in the United States, accounting for 40,000 deaths annually [1]. A great majority of cirrhosis patients have subclinical illness. As the condition worsens, they frequently decompensate quickly and are at a significant risk of morbidity, death, and a low quality of life [2]. The predictive algorithm for the end-stage sodium (Na) score is a simplified logistic regression (LR) model created in 2002 that is used to predict mortality. It can assist with triage, therapy, and monitoring, accurately forecasting 90-day mortality at high scores [3].

Risk prediction scores are frequently used to inform treatment choices ranging from anticoagulation to cholesterol reduction, to life-sustaining critical care [4]. Although their predictive effectiveness is restricted, the most popular scores incorporate a small number of readily measured factors, enabling transparent calculation and interpretability [5].

Machine learning (ML) techniques can help with prognostication. The accuracy of these methods is increased by using many interactions and a wide variety of predictions in a nonlinear pattern. However, its implementation is hampered in the most sophisticated informatics environments by its numerous variables and the intricacy of scoring systems [6]. Despite the help of informatics infrastructure, the algorithms' "black box" character renders them incomprehensible to patients and clinicians. Optimizing the trade-off between accuracy and interpretability and facilitating later adoption are two potential benefits of a hybrid approach that leverages machine learning's strengths to provide more straightforward, clinically explicable risk assessments [7].

Machine learning (ML) distinguishes itself from conventional predictive models by identifying complex data patterns that enhance prediction outcomes [6]. Conventional methods use basic models with limited variable ranges, while machine learning techniques' vast processing capacity yields accurate predictions that are dynamic and detailed. Complex interpretation issues and strict data requirements may arise for connected medical applications that use machine learning. Machine learning serves as an excellent tool for forecasting patient mortality from cirrhosis because of the complex disease linkages and unobtainable patterns [3].

Despite its promise, the integration of ML in clinical practice is accompanied by challenges, such as model interpretability, data quality, and generalizability across different healthcare settings [8]. Moreover, the lack of a systematic evaluation of existing studies hinders the comprehensive understanding of the predictive power of ML in cirrhosis mortality. Addressing these gaps is essential to facilitate the translation of ML models from research to clinical application.

This systematic review aims to evaluate the current state of evidence on the predictive capabilities of ML in estimating mortality risk among cirrhosis patients. By synthesizing findings from published studies, this review seeks to highlight the strengths, limitations, and future directions of ML-based approaches in this critical area of hepatology. Through this, we hope to provide valuable insights for researchers, clinicians, and policymakers seeking to enhance the management of cirrhosis through data-driven solutions.

## Review

Methodology

Study Design

This systematic review was conducted following the Preferred Reporting Items for Systematic Reviews and Meta-Analyses (PRISMA) guidelines [9].

Eligibility Criteria

Peer-reviewed original research articles that used machine learning techniques and created or verified tools, ratings, or programs for predicting death in cirrhosis patients were considered. Research studies predicting fatalities in severe liver failure, which utilized image-based variable inputs, were disqualified. The inclusion and exclusion criteria are given in Table 1 in detail.

**Table 1:** Eligibility criteria for the inclusion and exclusion of studies MELD, model for end-stage liver disease; AUC, area under the curve

Category	Inclusion Criteria	Exclusion Criteria
Population	Adults diagnosed with cirrhosis	Studies involving non-cirrhosis-related conditions
Intervention/exposure	Application of machine learning models for predicting mortality in cirrhosis patients	Studies without the use of machine learning techniques or models
Comparison	Traditional prognostic models (e.g., Child-Pugh and MELD), if available	Studies without comparator models, where applicable
Outcomes	Mortality prediction accuracy, including metrics such as AUC, sensitivity, and specificity	Studies that do not report mortality prediction outcomes or related performance metrics
Study design	Original research articles, including observational studies, cohort studies, and cross-sectional studies	Reviews, editorials, conference abstracts, case reports, or non-original research articles
Language	Studies published in English	Studies published in languages other than English
Publication type	Peer-reviewed articles	Grey literature (unless high-quality), unpublished data, or preprints not peer-reviewed

Search Strategy

In December 2024, we carried out a comprehensive, systematic search for pertinent studies across four databases: Scopus, Web of Science, PubMed, and the Cochrane Library. Without regard to the publishing date, we only look for research that has been published in English. Every reference was gathered and kept in the EndNote X9 Library (Clarivate, Philadelphia, PA). When the same reference is gathered from a different database, EndNote X9 automatically eliminates the duplicates. To achieve the goal of this systematic review, searches were expanded to include papers from prior examinations of the reference lists of pertinent articles. The search strategy combined keywords and Medical Subject Headings (MeSH) terms related to cirrhosis, mortality prediction, and machine learning. Example keywords included "cirrhosis", "liver disease", "mortality", "machine learning", "artificial intelligence", "predictive modeling", and their synonyms. Boolean operators (AND and OR) and truncations (*) were applied to maximize the search results. The detailed search strategy for each database is provided in the Appendices.

Study Selection

All identified studies were imported into reference management software, and duplicates were removed. Three authors (HAAA, HBA, and MEAI) of our study serve as reviewers for study selection. Two independent reviewers (HAAA and HBA) screened the titles and abstracts for relevance. Full-text articles of potentially eligible studies were retrieved and assessed against the inclusion criteria. Disagreements were resolved through discussion or consultation with a third reviewer (tiebreaker) (HBA).

Quality Assessment and Data Extraction

Two researchers independently assessed the studies and categorized them by publication year, first author names, and experimental models. They used a standardized form to capture individual data on study characteristics, methods, and outcome measures. Disagreements over the selection of studies and data extraction were initially resolved through discussions between the two primary reviewers. If consensus could not be reached after these discussions, a third reviewer was consulted to mediate and provide a final decision. This approach ensured a transparent and unbiased resolution process and improved the reliability of the study selection and data extraction steps. Finally, the methodological quality of the included studies was assessed using six correction scales.

Results

Search Results

**Figure 1:**
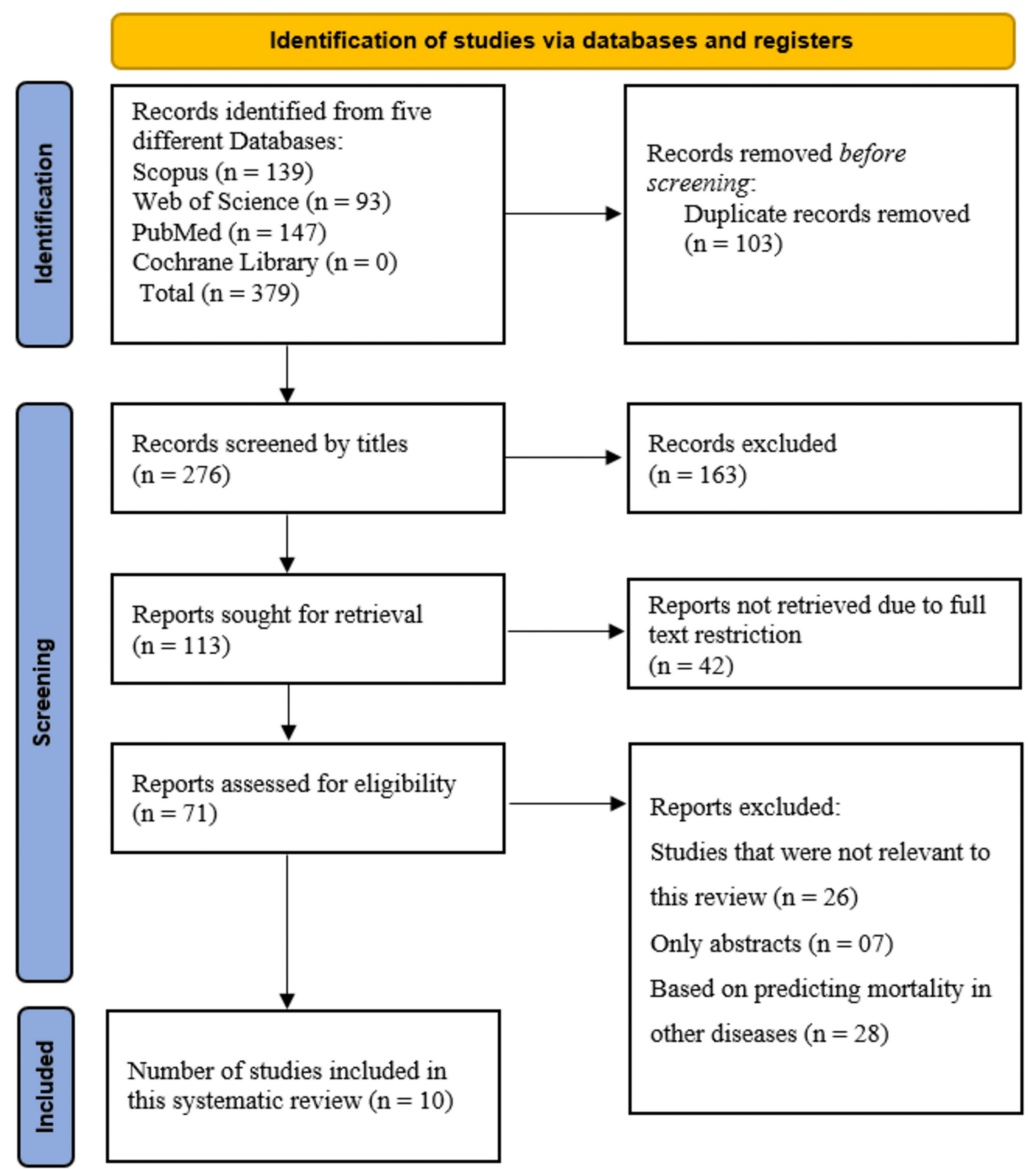
PRISMA flowchart PRISMA: Preferred Reporting Items for Systematic Reviews and Meta-Analyses

We located 379 different studies on these databases. Upon extracting all the studies to the EndNote library, 103 were removed as duplicates. One hundred sixty-three titles that had nothing to do with our study were removed after the titles for the remaining 276 papers were reviewed. In order to ascertain whether the full-text articles were available, we examined the remaining 113 research. Forty-two of these research studies were disqualified due to open-access constraints. Last but not least, 61 full-text papers out of the remaining 71 were rejected; 26 of them were deemed irrelevant, seven were abstracts only, and 28 were centered on ML in other illnesses. This systematic review included 10 full-text articles (Figure 1).

Characteristics of Included Studies

Ten retrospective studies were included. Four of these were carried out in Taiwan [10-13], while two were carried out in the United States [7,14]. The final four investigations were carried out in Germany [15], Italy [16], China [17], and India [18]. Five out of 10 studies were single-centered. Studies focusing on liver cirrhosis individuals and those waiting for liver transplantation were included in our analysis; these studies covered a range of death forecasts in liver disease, including two on 30-day fatalities, four on three-month fatalities, and four on one-year mortality. Table 2 provides a summary of the features of the included studies.

**Table 2:** Summary of studies included in this systematic review Internal validation: assesses a model's performance using the same dataset or a subset of the dataset it was trained on. External validation: tests a model's performance on an entirely independent dataset to evaluate its generalizability NASH, nonalcoholic steatohepatitis; ANN, artificial neural network; AUROC, area under the receiver operating characteristic; CART, classification and regression tree; DNN, deep neural network; GDB, gradient boosting decision; GBM, gradient boosting machine; LASSO, least absolute shrinkage and selection operator; LDA, linear discriminant analysis; LR, logistic regression; NPV, negative predictive value; PPV, positive predictive value; RFC, random forest classifier; RBF; radial basis function; SVM, support vector machine

Authors	Study Location	Study Design	Sample Size	Centers	Cause of Liver Disease	Model(s) Evaluated	AUROC	Sensitivity	Specificity	PPV	NPV	Accuracy
Cucchetti et al. [16]	Italy and the United Kingdom	Retrospective cohort	251	Multi	Chronic liver disease caused by alcohol, cholestatic liver disease, and viral cirrhosis	ANN	External, 0.96; internal, 0.95	68%	95%	67%	95%	91%
Gibb et al. [15]	Leipzig	Retrospective cohort	654	Single	Sclerosing cirrhosis, biliary cirrhosis, autoimmune hepatitis, alcohol, hepatitis B/C, and unidentified etiology (NASH and cryptogenic cirrhosis)	Penalized regression	0.961	Not reported	Not reported	Not reported	Not reported	Not reported
Guo et al. [10]	Taiwan	Retrospective cohort	34,575	Single	Hepatitis C, alcoholic cirrhosis, nonspecific cirrhosis, congestive cirrhosis, biliary cirrhosis, Wilson's disease, esophageal varices, hepatorenal syndrome, bacterial peritonitis, and pigmentary cirrhosis	ANN model (various timeframes)	Three months, 0.87; six months, 0.88; one year, 0.85	Not reported	Three months, 0.92; six months, 0.89; 365 days, 0.84	Not reported	Not reported	Three months, 0.90; six months, 0.86; one year, 0.83
Hu et al. [14]	The United States	Prospective	2,170	Multi	Alcohol, hepatitis C, and fatty liver (nonalcoholic)	LR, RFC, and RBF kernel SVM	LR, 0.67; SVM, 0.58; RFC, 0.62	Not reported	Not reported	Not reported	Not reported	Three-month mortality: LR, 0.84; RFC, 0.85; SVM, 0.84
Kanwal et al. [7]	The United States	Retrospective cohort	107,939	Multi	Not reported	GDB, LASSO regression, partial path logistic model	GDB, 0.81; LASSO, 0.78; partial path, 0.78	Not reported	Not reported	Not reported	Not reported	Not reported
Lin et al. [11]	Taiwan	Retrospective cohort	1,214	Multi	Chronic liver conditions with or without complications	Random forest (RF), AdaBoost, LDA, SVM, naive Bayes, and CART	RF, 0.852; AdaBoost, 0.833	LDA, 0.70; naive Bayes, 0.29; SVM, 0.31; AdaBoost, 0.45; CART, 0.37; RF, 0.37	CART, 0.37; LDA, 0.82; RF, 0.36; AdaBoost, 0.44; SVM, 0.96; naive Bayes, 0.93	Not reported	Not reported	SVM, 0.81; LDA, 0.82; naive Bayes, 0.78; RF, 0.82; AdaBoost, 0.81; CART, 0.79
Yu et al. [12]	Taiwan	Retrospective cohort	932	Single	Nonmalignant chronic liver disease	Random forest (RF) and AdaBoost	RF, 0.838; AdaBoost, 0.792	Not reported	Not reported	Not reported	Not reported	Not reported
Simsek et al. [13]	Taiwan	Retrospective cohort	124	Single	Cryptogenic, chronic hepatitis B or C, vascular disease, and alcoholic liver disease	LightGBM	One month, 0.86; three months, 0.85; one year, 0.76	Not reported	Not reported	Not reported	Not reported	Not reported
Yu et al. [17]	China	Prospective	932	Single	Non-cholestatic cirrhosis	XGBoost, LR, DNN, GBM, RF, and stacking	GBM, 0.888; XGBoost, 0.886; LR, 0.673; stacking, 0.85; DNN, 0.83; RF, 0.866	Not reported	Not reported	Not reported	Not reported	Not reported
Banerjee et al. [18]	India	Retrospective cohort	92	Multi	Cirrhosis	ANN	External, 0.84; internal, 0.95	External, 0.89; internal, 0.90	External, 0.92; internal, 0.92	External, 0.59; internal, 0.90	External, 0.97; internal, 0.92	External, 0.91; internal, 0.90

The models were trained using varied sample sizes, with a median of 793 and a range of 124-107,939 samples. Six of the cited studies used datasets with a mean of 329 samples smaller than 1,000. Two studies used datasets with an average of 2,036 samples, which is less than 5,000 samples. A substantial dataset of 34,570 samples was employed in one study; however, only one study used a dataset of more than 100,000 samples, exactly 107,939. There was a comparatively small use of other data for the validation of models among the research that were cited since just three of them used external datasets.

Comparison of Machine Learning Models

All the included studies employed supervised machine learning. Five studies used gradient descent boosting, random forest (RF), and logistic regression (LR). Four studies employed artificial neural network (ANN). Two research studies each used a classification and regression tree (CART) and support vector machine (SVM). Only one study used linear discriminant analysis, stacking, and naive Bayes.

Performance Metrics

Nine studies revealed that ML models performed better than classical MELD-Na, MELD, or Child-Pugh's model in the comparison analysis, while one study found that classical models and ML models performed similarly. Banerjee et al.'s ANN outperformed Child-Pugh's score of 0.55 with an accuracy of 0.90 [18]. Cucchetti et al.'s ANN fared better than MELD, with an area under the curve (AUC) of 0.96 as opposed to 0.86 [16]. With an AUC of 0.88, Guo et al.'s ANN outperformed MELD's 0.81 [10]. Lin et al.'s RF model outperformed MELD's with a score of 0.85 [11]. MELD-Na scored 0.65, and Yu et al.'s RF model produced 0.83 [12]. Gibb et al.'s LR outperformed MELD's 0.934 with an AUC of 0.96 [15]. The AUC of 0.81 for Kanwal et al.'s gradient boost was noticeably higher than MELD's 0.67 [7]. MELD-Na received a score of 0.73, while Simsek et al.'s gradient boost obtained an AUC of 0.85 [13]. The results of Yu et al.'s gradient boosting machine (GBM) were 0.886, higher than MELD-Na's 0.782 [17]. Interestingly, Hu et al.'s study was the only one to demonstrate that LR performed similarly to MELD-Na, with an average accuracy of 0.71 versus 0.72 [14].

Methodological Quality Assessment

The quality of the included studies was evaluated based on six criteria: (A) publication in a peer-reviewed journal, (B) random group allocation, (C) blinded outcome assessment, (D) sample size calculation, (E) adherence to animal welfare regulations, and (F) the disclosure of potential conflicts of interest.

The included studies' methodological quality scores ranged from 4 to 5 out of a possible 6 points. Specifically, four studies (40%) scored 4 points, and six studies (60%) scored 5 points. No study scored below 4 points. Table 3 provides a detailed summary of the methodological quality assessment.

**Table 3:** Methodological quality assessment of included studies using the Jadad tool (A) Publication in a peer-reviewed journal, (B) random group allocation, (C) blinded outcome assessment, (D) sample size calculation, (E) adherence to animal welfare regulations, and (F) the disclosure of potential conflicts of interest A score of 4 indicates that the study met four of the six criteria. A score of 5 indicates the study met five of the six criteria

Study	A	B	C	D	E	F	Total
Cucchetti et al. [16]	√	√	_	_	√	√	4
Gibb et al. [15]	√	√	_	√	√	√	5
Guo et al. [10]	√	√	_	√	√	√	5
Hu et al. [14]	√	√	_	√	√	√	5
Kanwal et al. [7]	√	√	_	√	√	_	4
Lin et al. [11]	√	√	_	√	√	√	5
Yu et al. [12]	√	√	√	_	√	_	4
Simsek et al. [13]	√	√	_	√	√	_	4
Yu et al. [17]	√	√	_	√	√	√	5
Banerjee et al. [18]	√	√	_	√	√	√	5

Discussion

Our review highlights how ML models might improve end-stage liver disease (ESLD) patient death prediction. With an area under the receiver operating characteristic (AUROC) score ranging from 0.71 to 0.96, our results highlight the need for systematic validation processes and customized dataset management to guarantee the clinical usability and dependability of ML in medical applications. The inherent complexity of predicting ESLD outcomes, the vulnerability to overfitting, and important data quality factors are highlighted by the observed performance disparities, such as those between models based on small versus big datasets. In order to provide clinical practitioners with more confidence and enhance interpretability, the paper highlights the necessity for clear and understandable artificial intelligence (AI) systems [19].

The complex and nonlinear relationships between biological components make mortality prediction extremely difficult [20]. Prediction models that have historically been essential to assessing ESLD outcomes include the MELD-Na, MELD, and Child-Pugh scores. Nevertheless, these models' simplicity frequently masks the intricate relationships between variables that are essential for precise mortality prediction [21]. By utilizing a variety of parameters, machine learning models, on the other hand, provide a clear advantage by improving the accuracy of mortality prediction and revealing intricate nonlinear relationships [22]. According to this review, which is consistent with earlier research, machine learning models have the potential to improve predictive accuracy by accommodating and understanding complex nonlinear relationships between input variables [23].

However, there are certain issues with using ML in clinical settings. The generalization of the models in our research population is hampered by the dependence on small datasets due to the restricted availability of data. Furthermore, a major barrier to the clinical adoption of ML methods is their lack of interpretability and transparency [24]. ANNs and other machine learning techniques need a lot of data and can overfit the training dataset. Overfitting is a possibility in studies with small sample sizes, especially those with less than 100 samples, and insufficient external validation. Despite their promise for better performance on massive data, ANNs remain ineffective in ESLD mortality estimation, as only a small percentage of research (4/10) used them. Additionally, seven out of 10 studies did not apply any external validation. The generalizability of such ML models and our comprehension of patient mortality risk may both benefit from a consistent external dataset containing a balanced and heterogeneous population [25].

The general "black box" character of ML models necessitates a change to more interpretable and explicable models in order to get broader clinician acceptance [26]. One study attempted to better explain the significant factors of the underlying machine learning models by using graphics [12]. In a similar way, Kanwal et al. picked a basic ML model with lower accuracy but better transparency over other more advanced but transparent ML models [7]. This idea is supported by a growing body of research that calls for more straightforward, interpretable models in order to improve explainability [27]. Furthermore, adding socioeconomic and psychosocial factors to machine learning models may result in a more comprehensive, nuanced understanding of patient outcomes. This method not only solves the shortcomings of predictive models that primarily rely on clinical and laboratory data but also fits with the growing emphasis on including broader factors of health into these models [28].

Our systematic review emphasizes how ML models can improve the prediction of ESLD mortality. However, issues with the integrity of data and model transparency must be resolved if this potential is to be fully realized in clinical practice. One crucial issue with these studies is the lack of clarity surrounding the effect of missing data. Systematic bias may result from missing data, especially if specific patient groupings lack representation in the medical system. All studies in our systematic review took into consideration base factors including age, gender, and ethnicity; only a small percentage (3/10) handled and specified missing data as a component of their research. This negligence may lead to biases in machine learning algorithms, which can be caused by misclassification, measurement error, and sample size [29]. In order to successfully go from research to real-world clinical application, studies should concentrate on creating reliable, interpretable models that have been verified over a variety of datasets and taking into account a wide range of predictive variables. Gaining physician trust and guaranteeing dependable deployment in a range of clinical settings depend on transparent models that offer precise, useful insights. Leading institutions' use of ML techniques demonstrates the increasing awareness of their potential to improve healthcare decision-making [30].

Recommendations

We encourage upcoming researchers to openly publish their data and code so that peer reviews can find any overfitting. Furthermore, to learn more about the practical effectiveness of these models, pragmatic trials that evaluate therapies in standard clinical settings are crucial. By overcoming these obstacles, machine learning models can greatly enhance the predictive tools offered by ESLD, giving physicians advanced tools to improve patient care and results. This represents a substantial shift from static, traditional models to dynamic, data-driven approaches.

Limitations

A major issue with these studies is the lack of clarity regarding the significance of missing data. Systematic bias may result from missing data, especially if specific patient groupings have little representation in the medical system.

## Conclusions

Our review concludes by highlighting the potential of ML techniques to enhance the prediction of liver cirrhosis mortality. However, future research should concentrate on creating reliable, interpretable models that are validated against various datasets and incorporate a wide range of predictive variables in order to facilitate an efficient transition from studies to the bedside. Furthermore, code sharing and data transparency will make it easier for peer reviews to spot any model bias. By tackling these issues, machine learning models have the potential to significantly improve the instruments for predicting outcomes in ESLD, signaling a significant departure from conventional, straightforward models in favor of more dynamic, data-driven strategies.

## Appendices

Table 4 shows the detailed search strategy for each database. 

**Table 4:** Search strategy for each database

Serial Number	Database	Search Strings
1	Scopus	("machine learning" OR "artificial intelligence" OR "ML" OR "AI") AND ("cirrhosis" OR "liver cirrhosis") AND ("mortality" OR "death" OR "survival") AND ("predict" OR "prediction" OR "prognosis") AND ("model" OR "algorithm")
2	Web of Science	TS=("machine learning" OR "artificial intelligence" OR "ML" OR "AI") AND TS=("cirrhosis" OR "liver cirrhosis") AND TS=("mortality" OR "death" OR "survival") AND TS=("predict" OR "prediction" OR "prognosis") AND TS=("model" OR "algorithm")
3	PubMed	("machine learning"[Mesh] OR "artificial intelligence"[Mesh] OR "ML" OR "AI") AND ("cirrhosis"[Mesh] OR "liver cirrhosis") AND ("mortality"[Mesh] OR "death" OR "survival") AND ("predict"[Title/Abstract] OR "prediction"[Title/Abstract] OR "prognosis"[Title/Abstract]) AND ("model"[Title/Abstract] OR "algorithm”)
4	Cochrane Library	("machine learning" OR "artificial intelligence" OR "ML" OR "AI") AND ("cirrhosis" OR "liver cirrhosis") AND ("mortality" OR "death" OR "survival") AND ("predict" OR "prediction" OR "prognosis") AND ("model" OR "algorithm”)
